# Influence of exercises on patellar height in women with patellofemoral pain syndrome

**DOI:** 10.1590/1413-78522014220200748

**Published:** 2014

**Authors:** Lilian Ramiro Felicio, Ana Claudia Spechoto Camargo, Augusto do Prado Baffa, Débora Bevilaqua-Grossi

**Affiliations:** 1 Centro Universitário Augusto Motta, Rio de Janeiro, RJ, Brazil, Centro Universitário Augusto Motta, Rio de Janeiro, RJ, Brazil; 2 Universidade de São Paulo, Faculdade de Medicina de Ribeirão Preto, Ribeirão Preto, SP, Brazil, Faculdade de Medicina de Ribeirão Preto, Universidade de São Paulo, Ribeirão Preto, SP, Brazil

**Keywords:** Patellofemoral pain syndrome, Isometric contraction, Patella

## Abstract

**OBJECTIVE:**

To evaluate the patellar height of volunteers with and without patellofemoral pain syndrome (PPS) during maximal voluntary isometric contraction (MVIC) in open kinetic chain (OKC) and closed kinetic chain (CKC) exercises.

**METHODS:**

Twenty healthy women, and nineteen women with patellofemoral pain syndrome were evaluated and subjected to nuclear magnetic resonance imaging during rest and MVIC in OKC and CKC at 15°, 30°, and 45° knee flexion. The patellar height was assessed by the K-Pacs program,using the Insall-Salvati index. For each exercise and knee position, patellar height was measured three times and the procedure was repeated after seven days.

**RESULTS:**

These data did not confirm our hypothesis that OKC exercises promote increased patellar height.

**CONCLUSION:**

Patellar height is not associated with PPS and suggests that CKC exercises lead an increased patellar height during knee position at 15º and 45º flexion for the PPS group. ***Level of Evidence II, Comparative Prospective.***

## INTRODUCTION

Patellar high is a condition frequently observed in individuals with knee malfunctions, such as the patellofemoral dysfunction (PFD), a possible correlation with its development may be observed.^1^ ^,^ ^2^

Athletes can often present patellar high, particularly those who feel pain as a result of a specific knee dysfunction.^2^ These individuals show patellofemoral pain syndrome (PPS) as a major condition affecting the knee.^3^ ^,^ ^4^

The increase of patella height is related to an abnormal patellofemoral joint contact that leads to increased patellofemoral compressive forces, compared with lower patella and normal patellar height.^5^Furthermore, it is known that the patellar high is associated with a reduction of the contact area of ​the joint, possibly also contributing to increased articular stress.^6^

Understanding patellofemoral kinematics of individuals with PFD in open kinetic chain (OKC) and closed kinetic chain (CKC) exercises in all plans, including the sagittal plan, is of great importance, since these exercises are part of the athletes' sports training, as well as PPS conservative treatment, being effective in reducing symptoms.^7^ ^,^ ^8^

Some studies that studied, through magnetic resonance imaging of the frontal and sagittal plans, patellofemoral kinematics of individuals with PPS concluded that activity in early knee flexion, especially in OKC, can lead to an increase in lateral patellar displacement,^8^and that lateral patellar displacement and tilt are higher in maximum voluntary isometric contraction (MVIC) in OKC when compared to CKC. However, it is not established which is the influence of OKC and CKC exercises in patellar height in individuals with PPS.

Thus, the present study aimes to compare patellar height in healthy and PPS individuals during MVIC in OKC and CKC through magnetic resonance imaging 

Since patellar kinematics on the front plan is influenced by the type of exercise performed, the study has hypothesized that OKC exercises could lead to an increase in patellar height in individuals with PPS higher than those observed during CKC exercises.

## MATERIALS AND METHODS

A functional kinetic evaluation was performed in 39 sedentary female volunteers, who were subsequently divided into two groups: control group, which consisted of clinically healthy subjects (n = 20) and PPS group, composed of individuals with Patellofemoral Pain Syndrome (n = 19) according to the inclusion and exclusion criteria for each group. (Table 1) Anthropometric data of PPS volunteers and control groups are shown in Table 2.

**Table 1:** Inclusion and exclusion criteria to control and PFD groups.

	Control Group	PFD Group
Inclusion criteria	• Presence of at most two signals indicating misalignment of the lower limb in functional assessment (increased Q angle, excessive subtalar pronation, abnormal mobility of the patella);^12^ • Absence of pain recorded on the visual analogue scale (VAS) in the last month.^13^	• Presence of at least three signs indicating PPS observed in functional assessment; • Presence of pain of at least three cm visual analog scale (VAS) in the last month;^13 ^ • Presence of pain in at least two functional activities (e.g. climbing and descending stairs, squatting, kneeling, running).^9,12^
Exclusion criteria^12^	• History of injury or surgery on the osteomioarticular system of the hip, knee, or ankle; • Individuals with neurological, cardiovascular or rheumatic diseases, • Individuals with dislocation or subluxation of the patella.	

**Table 2:** Anthropometric data of individuals in control and PFD groups.

Parameters	Control (n=20)	PFD (n=19)
Age (years)	21.5 ± 2.16	23.47 ± 3.24
Height (cm)	160.75 ± 5.23	161.63 ± 2.24
Weight (Kg)	54.44 ± 5.23	57.89 ± 6.91

Student t test for independent samples, from the SAS program was used. The application of statistical tests showed no difference in variables between the groups.

All subjects were informed about the procedures to be performed and signed a disclosure and consent form, in accordance to the standards of the Ethics Committee on Human Research of Hospital das Clínicas, FMRP, USP (Process HCRP 4250/2005).

Volunteers from both control and PPS groups performed magnetic resonance imaging (MRI) during rest and MVIC in OKC and CKC in three different knee flexion angles: 15º, 30º, and 45º. The evaluation of the patellofemoral kinematics was performed on the dominant member in the control group, and the injured member in the PPS group. Throughout the examination verbal command was performed, in order to encourage the volunteers to maintain maximum effort during the examination.

The images were obtained by means of magnetic resonance imaging equipment (MRI) using a Siemens Magnetom Vision 1.5 T device (Erlangen, Germany) using a 51x21 cm knee coil, with its center aligned with the center of the patella.

Images were acquired with 15 msec repetition time (RT), 6 msec echo time (ET) 512x128 matrix and 7 mm slices thickness. The image in the sagittal plan was generated from the image in the axial plan with the greatest latero-lateral diameter among the six acquired images.^8^

### Procedures

**Figure 1:**
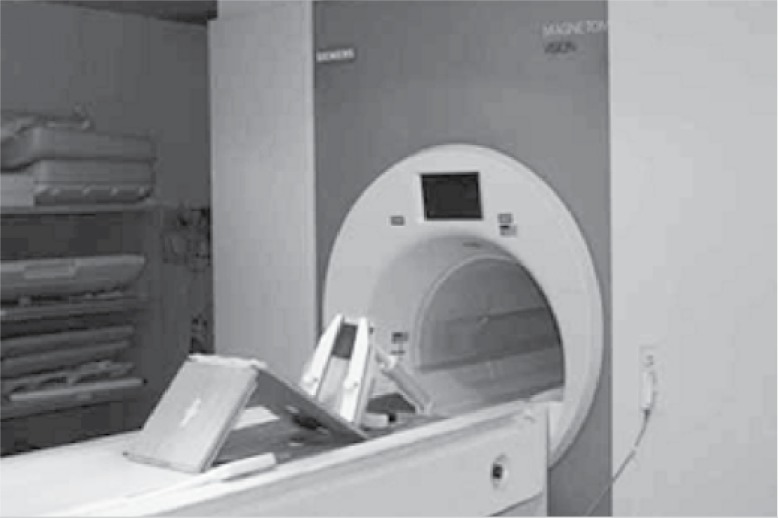
Adjustable and articulated wooden stand for knee positioning during MVIC at OKC and CKC.

During the MRI exam, patients remained in the supine position and knees were positioned at 15°, 30°, and 45° flexion using a goniometer (Carci^(r)^, Brazil) on a wooden support with non-metallic hinges to avoid interference in MRI images.^9^ The order in knee positioning, as well as the type of contraction performed were randomized. (Figure 1)

Velcro^(r)^ tapes were used around the legs to stabilize the hip, ankle and foot. The volunteers were verbally encouraged to strengthen while extending the knee (OKC) or push the support (CKC) and maintain it during six seconds of MVIC^10^ to perform the image in the sagittal plan. Between each activity there was two minutes resting time in order to prevent fatigue.

**Figure 2:**
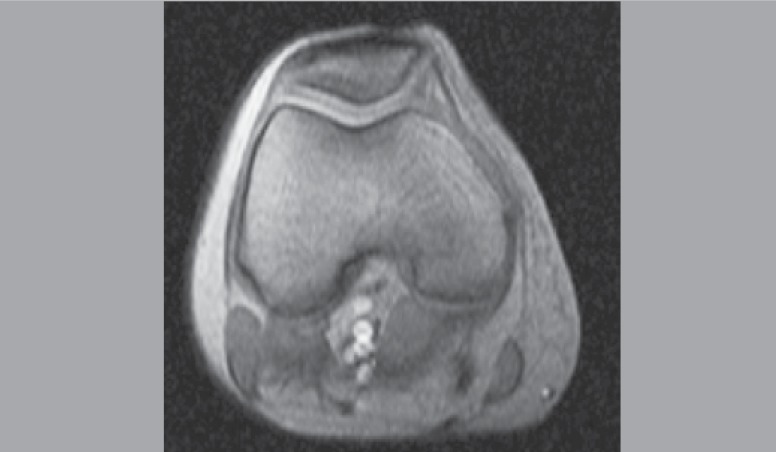
MRI Image of patella in axial plan with higher patellar latero- -medial diameter.

**Figure 3:**
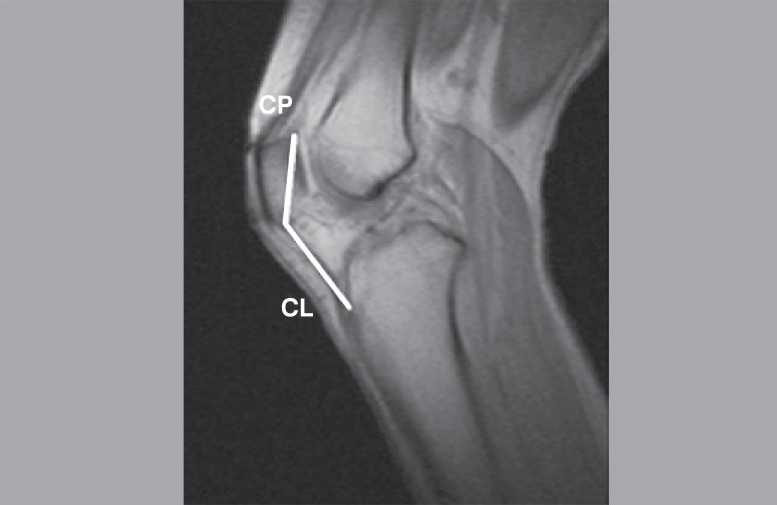
Sagittal view of the patellofemoral joint demonstrating the measurements of the ligament length (LL) and the longest diagonal patella length (PL) used in Insall-Salvati index.

The images in the axial plan of the patellofemoral joint used as reference were generated during rest and MVIC, in OKC and CKC for each knee angle, with a ratio of three seconds to generate each image in this plan. The image with higher lateral-medial patellar diameter^8^ was later selected (Figure 2) being the sagittal plan image generated from this one. The images were stored and analyzed using the K-Pacs software, version 1.6.0, using the Insall-Salvati index, which is the ratio between the length of the patellar ligament (LL) , measured from the lower pole of the patella to its insertion into the tibial tubercle, and the longest diagonal length of the patella (PL).^11^ (Figure 3) In this study the LL/PL ratios larger than 1.50 and less than 0.74 indicate, respectively, high and low patella.^12^

### Statistical Analysis

Three blinded measurements of patellar height were performed, and after seven days the measurements for analysis of intra-rater reliability were once again performed. The intraclass correlation coefficient (ICC 2,1) was used to this end. In order to compare the characteristics of the sample a student *t* test was used for independent measurements (p <0.05). The comparison of the average patellar height between the groups, type of exercise, and the knee angles was performed by ANOVA two way test, with a significance level of 5% (p <0.05). The program used to perform the statistical tests was the Statistical Analysis System (SAS).

## RESULTS

The analyzed parameters age, height and weight did not show significant differences between groups. Among the demographic and clinical parameters analyzed in the control group and the PPS group, only the values ​​of PPS questionnaire^11^ (control group, 99.0±2.3; PPS group, 77.9±8.8) and Q angle measurement (control group, 17.9±1.4; PPS group, 20.1±4.2) showed significant differences between groups. (Table 3)

**Table 3:** Mean and standard deviation of clinical parameters for the control group and the PPS group.

Parameters	Control (n=20)	PPS (n=19)
PFD questionnaire^14^ (0-100)	99.0 ± 2.3*	77.9 ± 8.8
Visual Analog Scale for pain during up and down step (0-100 mm)	___	22 ± 18
Duration of pain (months)	___	60.6 ± 27.2
Q Angle (degrees)	17.9 ± 1.4*	20.1 ± 4.2

Student t test was used (*p<0.05).

The patellar height measurements showed excellent levels of intra-rater reliability (ICC >0.75) for all situations and knee positions in both groups.

The mean of patellar height presented by both groups, at all knee flexion angles, OKC and CKC did not show values ​​greater than 1.5, indicating that both groups showed high patella during muscle contraction. Comparing patellar height between OKC and CKC exercises and between groups with and without pain, patellar height was not significantly different. (Table 4)

**Table 4:** Mean and standard deviation of Insall-Salvati index for control and PFD groups.

	Control Group			PPS Group		
Angle	Rest	OKC	CKC	Rest	OKC	CKC
15º	1,08 ± 0,11	1,19 ± 0,16^a^	1,18 ± 0,15^b^	1,07 ± 0,14	1,19 ± 0,15	1,22 ± 0,15^b^
30º	1,07 ± 0,15	1,21 ± 0,15^a^	1,19 ± 0,18^b^	1,09 ± 0,13	1,21 ± 0,17	1,21 ± 0,17
45º	1,13 ± 0,17	1,24 ± 0,19^a^	1,25 ± 0,21^b^	1,10 ± 0,19	1,19 ± 0,14	1,19 ± 0,16^b^

a significant difference between rest and maximal voluntary isometric contraction in OKC

b significant difference between rest and maximal voluntary isometric contraction in CKC

For the control group, the values ​​of patellar height showed significant differences between rest and MVIC in OKC and CKC at 15° (p<0.01), and between rest and MVIC in OKC and CKC at 30° and 45° (p<0.001). However, in PPS group, only CKC exercises at 15° and 45° knee flexion, significantly increased the patellar height compared to rest (p<0:01). (Table 4)

## DISCUSSION

Anthropometric data analyzed characterize a homogeneous sample. However, when analyzing clinical parameters related directly or indirectly with the PPS, such as PPS questionnaire proposed by Kujala *et al*.^13^ and Q angle, considered a risk factor for onset of pain, there is significant difference between the groups analyzed, agreeing with the data observed in other studies^14^ ^,^ ^15^.

The values of patellar height obtained by clinically healthy subjects and subjects with PPS showed no significant differences, when analyzed in different positions of the knee and in open and closed kinetic chain. These results indicate that the proximal patellar displacement during contractions in OKC and CKC did not differ between individuals with and without pain. Likewise, Felicio *et al.* ^9^ observed no difference in patellar kinematics, when analyzing other data such as patellar tilt between individuals with PPS and control individuals.

Despite possible biomechanical changes resulting from the patella may predispose to the onset of pain,^5^ our results are in agreement with other studies that found no significant differences in patellar height between individuals with and without knee pain,^14^ ^,^ ^16^ ^,^ ^17^ which suggest that the patellar high is not a predisposing factor for PPS.^17^

Although our study disagree with the results of Kannus,^18^ which associates pattelar high with development of patellofemoral dysfunction, currently the literature suggests that the predisposing factors of PPS include local factors related to the patellofemoral joint, such as increased patellar tilt and patellar height; distal factors related to changes in the ankle joint and foot and proximal factors related to changes in ankle and foot joint; and proximal factors related to hip alterations,^19^ and that local factors may not predispose to PPS,^20^ especially in individuals who show no patellar instability, as the sample studied in this work.

The fact that the control group presented patellar height increased in rest at MVIC in OKC and CKC indicates that muscle contraction causes proximal displacement of the patella, which is significant when compare to rest regardless the type of exercise performed. Thus, we note that in subjects with no pain complaints, the patella moves superiorly similarly to contractions in OKC and CKC, and that both types of exercises do not lead to high patella.

The most proximal patellar displacement found only on CKC at 15° and 45° knee flexion in individuals with PPS contradicts our hypothesis. These data show that although the lateral patellar tilt and displacement were higher in OKC in early knee flexion,^9^ proximal patellar displacement in the sagittal plan does not follow the same pattern, with this larger shift in CKC. Considering only the patellar height, we suggest that the two types of exercises, OKC and CKC, may be indicated in the conservative treatment of athletes or non-athletes with PPS, since these individuals do not have high patella or complaint of pain during exercise. Moreover, these exercises are widely used during sports training of athletes without pain symptoms.

## CONCLUSION

It should be considered in the completion of this work that, due to methodological limitations related to MRI equipment and the impossibility to use any metallic material in order to avoid distortions in images, control of the degree of effort through cell loads or dynamometers, was not performed during the period of the maximum effort.

Thus, according to the results of this study, it is concluded that patellar height is not a factor associated with the presence of PPS and it is suggested that both exercises are indicated since they do not promote significant proximal patellar dislocation in PPS patients.
